# Activation of the STING‐IRF3 pathway involved in psoriasis with diabetes mellitus

**DOI:** 10.1111/jcmm.17236

**Published:** 2022-02-17

**Authors:** Li Xiaohong, Zhang Zhenting, Yu Yunjie, Cai Wei, Xu Xiangjin, Xie Kun, Lin Xin, Lin Lu, Lu Jun, Chen Pin

**Affiliations:** ^1^ Fuzong Clinical Medical College of Fujian Medical University Fuzhou China; ^2^ Department of Endocrinology 900th Hospital of the Joint Logistics Team Fuzhou China; ^3^ Fujian Provincial Key Laboratory of Transplant Biology Laboratory of Basic Medicine Dongfang Hospital Xiamen University Fuzhou China

**Keywords:** mitochondrial damage, oxidative stress, psoriasis, STING‐IRF3 pathway, type 2 diabetes mellitus

## Abstract

Psoriasis and type 2 diabetes mellitus (T2DM) share similar inflammatory pathways in their pathogenesis. The stimulator of interferon genes (STING)‐interferon regulatory factor 3 (IRF3) pathway has recently been shown to play an important role in immune and metabolic diseases. In this study, we investigated the activation of the STING‐IRF3 pathway in human immortalized keratinocytes (HaCaT) cells treated with palmitic acid (PA) and imiquimod (IMQ). Additionally, we detected the STING‐IRF3 pathway in diabetic mice with imiquimod (IMQ)‐induced psoriasis and assessed the potential of STING inhibitor C‐176. Furthermore, skin samples from patients with psoriasis and diabetes were collected for immunohistochemical analysis. The results indicated that the STING‐IRF3 pathway was activated in HaCaT cells. Moreover, the STING pathway was also found to be induced in the skin tissue of diabetic mice with psoriasis; the inflammatory responses were ameliorated by treatment with C‐176. In the skin tissue samples of patients with psoriasis and diabetes, immunohistochemistry showed that the expression levels of STING and phosphorylated IRF3 were also significantly increased. Thus, we conclude that the STING‐IRF3 pathway is involved in the inflammatory response in the manifestation of psoriasis with T2DM. Inhibition of the activation of the STING pathway can ameliorate the development of psoriasis in diabetes and could be targeted for the development of therapeutic agents for these conditions.

## 1 INTRODUCTION

Pancreatic β‐cell dysfunction and insulin resistance (IR) are the main causes of type 2 diabetes mellitus (T2DM).^1^, ^2^ However, its pathogenesis has not yet been fully elucidated. Previous studies have shown that T2DM is also a low‐grade, chronic inflammatory disease. Pro‐inflammatory molecules can interfere with insulin signal transduction, cause immune dysfunction and trigger IR through oxidative stress.^3^, ^4^ Chronic inflammation, pancreatic β‐cell dysfunction and IR thus interact to promote the occurrence and development of T2DM.^5^

Psoriasis is a chronic immune‐mediated disease characterized by hyperproliferative keratinocytes and activated immune cell infiltration.^6^ The body's inflammatory response caused by microbial infection, trauma or metabolic disorders can activate dendritic cells to secrete tumour necrosis factor‐α (TNF‐α) and interleukin‐23 (IL‐23). Interleukin‐23 promotes T helper 17 (Th17) cell activation and proliferation. In turn, Th17 cells secrete interleukin (IL)‐17 and IL‐22 to induce keratinocyte proliferation.^7^, ^8^ Furthermore, as an autoimmune disease, psoriasis can trigger autoantigens such as cathelicidin LL‐37, melanocytic ADAMTSL5, lipid antigen PLA2G4D and keratin 17, leading to uncontrolled interferon (IFN) responses and driving complex autoimmune inflammation.^9^ The main clinical manifestations of psoriasis include skin erythema, scaliness and itchiness. These symptoms can severely reduce the quality of life of patients.^10^ Previous studies have suggested that psoriasis is related to metabolic syndromes such as obesity, dyslipidaemia, diabetes, hypertension and cardiovascular disease.^11^, ^12^

Psoriasis and IR have also been shown to be closely related.^13^, ^14^ Specifically, it has been shown that patients with diabetes and psoriasis have the same susceptibility gene loci, that is *PTPN22*, *ST6GAL1* and *JAZF1*.^15^ Elevated levels of inflammatory factors in psoriasis can then induce IR, which in turn contributes to the development of T2DM.^16^ The inflammatory response of the body activates the secretion of TNF‐α by dendritic cells and induces a variety of secondary mediators and adhesion molecules related to psoriasis. These pro‐inflammatory factors can also lead to IR. In particular, the high level of inflammation caused by activated Th17 cells leads to the production and aggravation of IR. In addition, our research group has confirmed that diabetes and psoriasis are closely related to inflammation through animal models and small sample clinical trials.^17^, ^18^ However, these pathways cannot fully clarify the pathogenesis of diabetes and psoriasis.

The stimulator of interferon genes interferon regulatory factor 3 (STING‐IRF3) pathway is a DNA‐sensing pathway located on the endoplasmic reticulum (ER) membrane. It can be activated by various stimuli, such as viral infection, ER stress and mitochondrial damage.^19^, ^20^ When mitochondrial damage occurs, mitochondrial DNA (mtDNA) then tends to be released into the cytoplasm and is recognized by the cytoplasmic DNA sensor cyclic GMP‐AMP synthase (cGAS) to activate STING. Activated STING is assembled with TANK‐binding kinase 1 (TBK1) to induce the activation of IRF3 and nuclear factor kappa B (NF‐κB) inflammatory pathways. The expression of interferon beta (IFN‐β) and other inflammatory cytokines such as TNF‐α, interleukin‐6 (IL‐6) and interleukin‐1 beta (IL‐1β) are ultimately upregulated.^21^, ^22^ Several studies have shown that the STING‐IRF3 pathway is closely associated with metabolic diseases.^23^, ^24^ However, the regulation of the STING‐IRF3 pathway in metabolic disorders and its mechanism still remains unclear.

In this study, we aimed to explore the role of the STING‐IRF3 pathway in psoriasis combined with T2DM. We observed this pathway in human immortalized keratinocytes (HaCaT) cells treated with palmitic acid (PA) and imiquimod (IMQ), animal models of psoriasis and T2DM, and skin samples from patients with psoriasis and T2DM.

## 2 MATERIALS AND METHODS

### 2.1 Cell culture and treatments

Human immortalized keratinocytes (HaCaT) cells were obtained from Shanghai Gefan Biological Cell Bank and cultured in Dulbecco′s Modified Eagle′s Medium (DMEM) with a high‐glucose content (HyClone), 10% foetal bovine serum (FBS) (Gibco) and 1% P/S at 37°C and 5% CO_2_. The HaCaT cells were divided into four groups: the normal control (NC) group, which was treated with culture medium containing only bovine serum albumin (BSA), and the three drug intervention groups. The first drug intervention group was cultured in media containing 2 mmol/L IMQ, the second group was cultured in media containing 0.3 mmol/L PA and the third group was cultured in media containing a combination of 2 mmol/L IMQ and 0.3 mmol/L PA. The supernatant from the treatment groups was collected, and total protein was extracted for the next experiment.

### 2.2 Animal models

All animal experiments were approved by the Animal Care and Ethics Committee of the 900th Hospital of the Joint Logistics Team (approval number: 2020‐059). Forty‐five 6‐week‐old male C57BL/6 mice were housed in cages under pathogen‐free conditions at 22–25°C and a 12‐h dark/light cycle. All mice had free access to food and water. After 1 week of adaptation, the mice were randomly divided into a normal chow diet (NCD) group (*n* = 18) and a high‐fat diet (HFD) group (*n* = 27). After 12 weeks on a HFD, mice in the HFD group were intraperitoneally injected with 40 mg/kg of streptozotocin (STZ) in citrate buffer (pH = 4.5), and mice in the NCD group were injected with the same amount of citrate buffer as a control. After 7 days, mice with fasting blood glucose (FBG) levels ≥11.1 mmol/L on three consecutive days were considered as T2DM models [hereafter referred to as the diabetes model (DM) group]. Thereafter, an intraperitoneal glucose tolerance test (IPGTT; 2 g/kg) was performed. Glucose levels in tail vein blood were measured continuously at 0, 30, 60, 90, 120 and 150 min post‐injection during the test. The area under the curve (AUC) was calculated according to the trapezoidal rule.

### 2.3 Experimental design

One week after STZ injection, mice were randomly divided into four groups (*n* = 6), two groups that received Vaseline ointment (NCD + Vaseline and DM + Vaseline), and two groups that received imiquimod (IMQ)‐induction (NCD + IMQ and DM + IMQ). A 3 × 4 cm^2^ area on the back of each mouse was shaved and treated with Vaseline ointment or IMQ cream (62.5 mg/animal) for seven consecutive days. After the experiment, the mice were sacrificed, and blood samples were collected from their hearts. Skin tissue was isolated from the anaesthetized mice and stored at −80°C, and a part of each tissue sample collected was fixed in paraformaldehyde for histopathological analysis as described in Section 2.5.

The other mice were randomly divided into three groups (*n* = 6): Vaseline ointment group (NCD + Vaseline) and IMQ‐induction group (DM + IMQ and DM + IMQ + C‐176). Before IMQ‐induced psoriasis‐like skin inflammation, the DM + IMQ + C‐176 group was injected with 7.5 μL (750 nmol) of C‐176 per mouse or DMSO dissolved in 85 μL corn oil once per day for 2 weeks. The dosage and time points of C‐176 were based on a previous study.^25^ The mice were then shaved and treated with IMQ cream or Vaseline ointment, as previously described.

### 2.4 PASI scores

An objective scoring system was established based on the clinical psoriasis area and severity index (PASI) to measure the degree of inflammation of the back skin during the construction of a psoriasis skin lesion model in mice. Erythema, scaling and thickening were evaluated independently, following methods described previously.^17^

### 2.5 Histopathological section and HE staining

The skin tissues of the mice were fixed with 4% paraformaldehyde, dehydrated and embedded in paraffin wax, then cut into 4‐μm‐thick sections, after which the sections were stained with haematoxylin and eosin (HE).

### 2.6 Immunohistochemistry

Skin sections (4 μm) were dewaxed, dehydrated, heat‐processed in citrate saline buffer for antigen retrieval and incubated with 3% H_2_O_2_ at 37°C for 10 min. Subsequently, incubation with 10% normal goat serum (ZSGB‐Bio) was performed at 37°C for 30 min. Then, the sections were incubated overnight at 4°C with primary antibodies (Table S1–1). The next day, the sections were incubated with biotin‐labelled secondary antibody working solution (ZSGB‐Bio) at 37°C for 30 min. Finally, the sections were stained with 3, 3′‐diaminobenzidine (DAB) and observed under a light microscopy. The relative positive staining index was measured according to staining intensity and positive area.^26^ The stained sections were analysed independently by two pathologists who were blinded to the experimental data.

### 2.7 Lipid peroxidation assay

Malondialdehyde (MDA) is considered a biomarker of oxidative stress. In the lipid peroxidation assay, MDA in the sample reacts with thiobarbituric acid (TBA), and a red‐coloured adduct MDA‐TBA is formed with a maximum absorbance at 532 nm. The absorbance of the MDA working solution was thus detected and analysed according to the manufacturer's instructions. The level of MDA is presented as µmol/mg protein.

### 2.8 Real‐time quantitative PCR

Total RNA from skin tissue samples was extracted using TRIzol reagent (Invitrogen), and the RNA was reverse transcribed into cDNA using a RevertAid cDNA synthesis kit (Thermo Fisher Scientific). The primers were synthesized by Fuzhou biogentechnology Co., Ltd. Quantitative polymerase chain reaction (qPCR) analysis was performed using a SYBR Green qPCR kit (Thermo Fisher Scientific) and quantification was normalized to the expression level of α‐actin. The primer sequences of the mouse genes are shown in Table S2.

### 2.9 Western blotting

Skin tissue samples of mice and HaCaT cells were lysed using radioimmunoprecipitation assay (RIPA) buffer (Beyotime). The protein lysates were then centrifuged at 14000 g at 4°C for 20 min. The bicinchoninic acid (BCA) method (Beyotime) was subsequently used to determine the total protein concentration in the lysates. The protein samples were separated on 10% SDS‐polyacrylamide gels and transferred to 0.45‐µm polyvinylidene difluoride membranes (Millipore), blocked with 5% BSA (Sangon Biotech) for 1 h and incubated with a specific primary antibody (Table S1–2) overnight at 4°C. Finally, the membranes were incubated with the secondary antibody at 37°C for 1 h. Enhanced chemiluminescence (ECL) imaging was performed using an ChemiScope 6000 Touch Integrated Chemiluminescence Imaging System (Clinx Science Instruments). Relative protein levels were analysed using the ImageJ software.

### 2.10 Enzyme‐linked immunosorbent assay

The mouse serum and HaCaT cell culture media of each group were further analysed to determine the concentrations of interferon gamma‐induced protein 10 (CXCL10) and IFN‐β using ELISA kits, according to the manufacturer's instructions (Elabscience) (Mouse IFN‐β ELISA Kit: E‐EL‐M0033c, Mouse CXCL10 ELISA Kit: E‐EL‐M0021c, Human CXCL10 ELISA Kit: E‐EL‐H0050c). Absorbance was measured at 450 nm using an Multiskan™ GO ELISA plate reader (Thermo Fisher Scientific) and the relative protein concentrations were calculated.

### 2.11 Human sample analysis

Approval to collect human skin samples was obtained from the Ethics Committee of the 900th Hospital of the Joint Logistics Team (approval number: SC‐2017‐007). Participants were recruited from the dermatology and diabetology clinics of the 900th Hospital of the Joint Logistics Team from December 2017 to June 2020. Twenty‐two participants were recruited for this study (Table S3). The inclusion and exclusion criteria are detailed in a previous study.^18^ Written informed consent was obtained from all recruited participants.

### 2.12 Statistical analysis

The data in our study are presented as the mean ± SEM. All statistical analyses were performed using Image‐Pro Plus 6.0 and GraphPad Prism 8 software. One‐way ANOVA or the unpaired Student's *t*‐test was used to compare the differences between the two groups. Statistical significance was set at *p* < 0.05.

## 3 RESULTS

### 3.1 STING‐IRF3 pathway involvement in cellular model of diabetes and psoriasis

**FIGURE 1:**
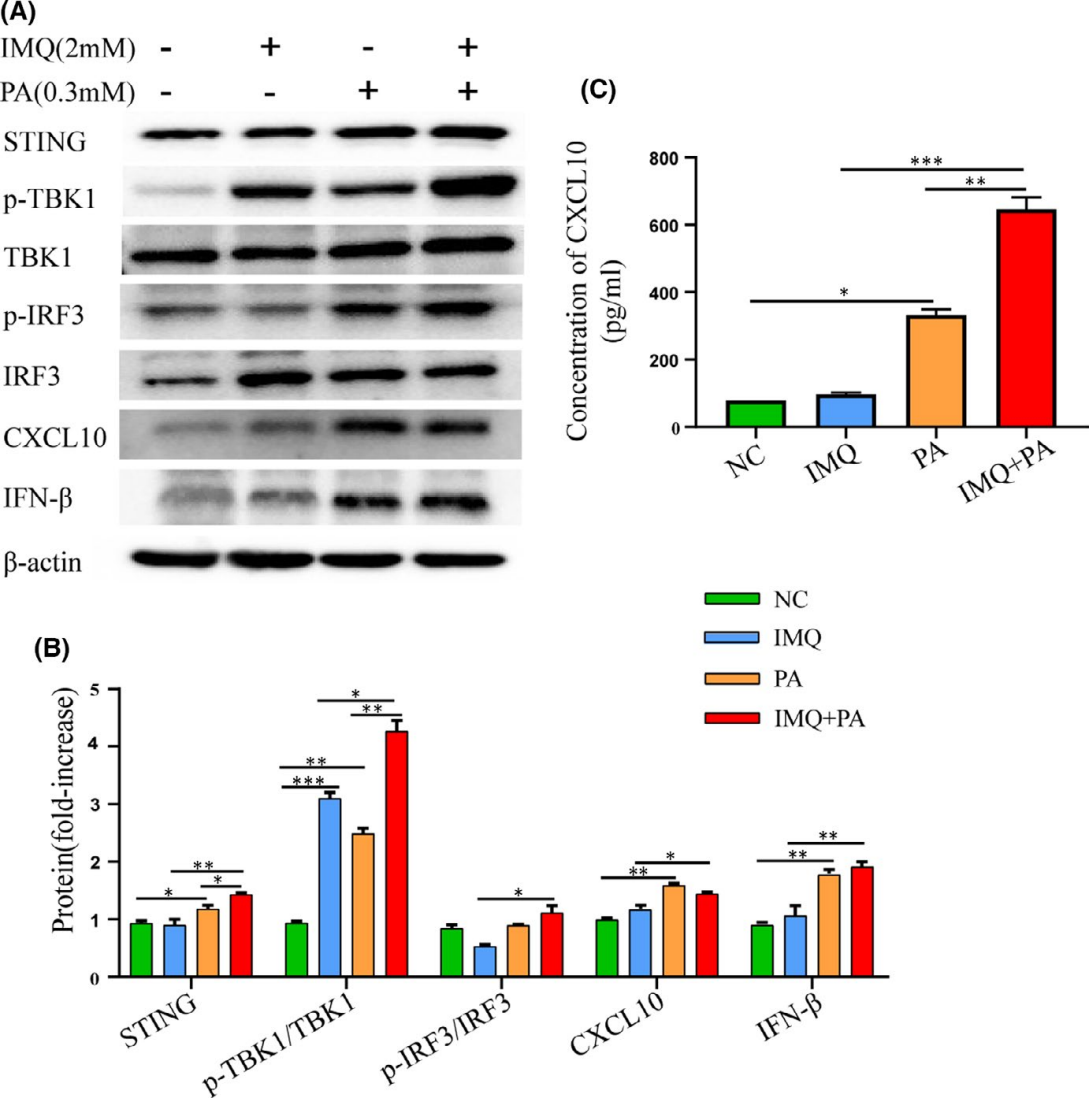
STING‐IRF3 pathway involvement in cellular model of diabetes and psoriasis. (A, B) Protein levels of STING, p‐TBK1/TBK1, p‐IRF3/IRF3, CXCL10 and IFN‐β were measured by Western blotting. (C) The expression level of CXCL10 in HaCaT cells was measured by ELISA. Data are represented as means ± SEM. *p < 0.05; **p < 0.01; ***p < 0.001; ****p < 0.0001

The STING‐IRF3 pathway is activated by PA and IMQ in HaCaT cells. Western blot analysis showed that the levels of STING, p‐TBK1/TBK1, p‐IRF3/IRF3 and IFN‐β were higher in the PA + IMQ group (Figure 1A,B). The results of ELISA showed that the secretion of CXCL10 was significantly upregulated in the PA + IMQ group (Figure 1C). These data indicate that the STING‐IRF3 pathway is involved in the inflammation induced by PA and IMQ in HaCaT cells.

### 3.2 Clinical parameters of psoriasis and T2DM animal model

**FIGURE 2:**
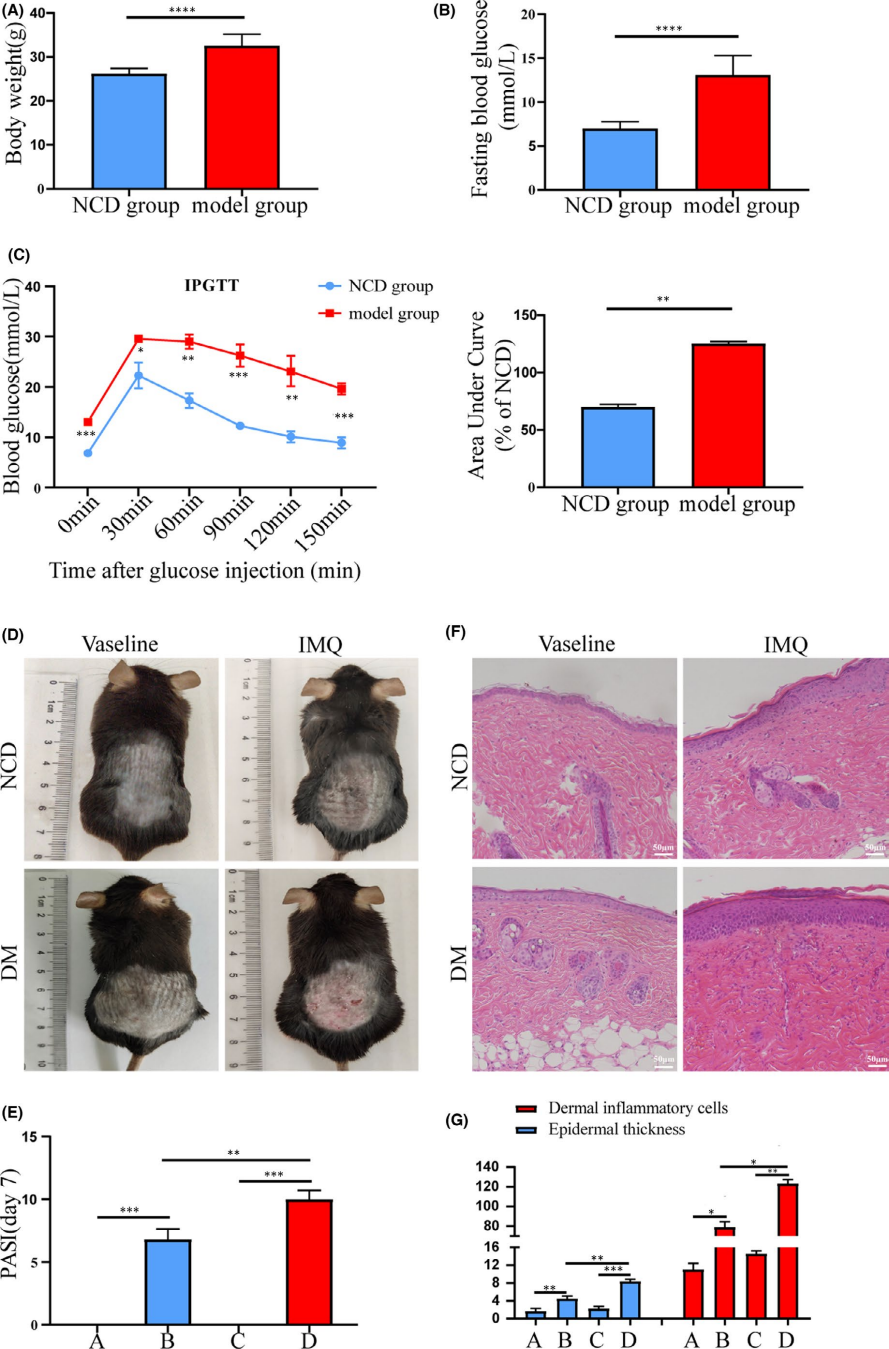
Clinical parameters of psoriasis and T2DM animal model. Group A, NCD + Vaseline, Group B, NCD + IMQ, Group C, DM + Vaseline, Group D, DM + IMQ. T2DM mouse model was induced by high‐fat diet (HFD) combined with low‐dose streptozotocin (STZ), the body weight (A) and fasting blood glucose (B) of mice in the NCD group were compared. Intraperitoneal glucose tolerance test (IPGTT) (2 g/kg) was evaluated by measuring the blood glucose levels of the tail vein and area under the curve (AUC) (C). After 7 days of intervention with IMQ, (D) visual changes in the skin on the back of each group, (E) comparison of PASI scores of back skin of mice in each group. (F) Histopathological changes were observed under light microscope after HE staining (×200). (G) The epidermal thickness and the number of inflammatory cells in the dermis were analysed by Image‐Pro Plus 6.0 software. Data are represented as means ± SEM (*n* = 6). *p < 0.05; **p < 0.01; ***p < 0.001; ****p < 0.0001; Scale bars, 50 µm

Body weight (BW) and FBG levels of mice were measured after diabetes was induced by a HFD combined with a low dose of STZ. The BW and FBG levels of mice in the DM group were markedly higher than those in the NCD group (Figure 2A,B). The IPGTT (2 g/kg) showed that the mice in the NCD group could tolerate glucose, whereas the DM group had impaired tolerance. The AUC analysis corroborated this conclusion (Figure 2C). These data indicate that we successfully established a mouse model of T2DM. The psoriasis model was induced after continuous intervention with IMQ for 7 days, and the mice showed obvious erythema, scaly skin and skin thickening. The severity of psoriasis lesions in the DM + IMQ group was markedly higher than that in the NCD + IMQ group. Further analysis showed that PASI was higher in the DM + IMQ group (Figure 2D,E). Haematoxylin and eosin staining showed that the epidermal tissues of the mice were thickened, the keratinization layer and cell layer were markedly increased, and the infiltration of inflammatory cells was also increased. These symptoms were more severe in the DM + IMQ group (Figure 2F). Further analysis showed that the epidermal thickness and the number of dermal inflammatory cells were markedly higher in the DM + IMQ group than in the NCD + IMQ group (Figure 2G). These data indicate that diabetes can aggravate psoriatic skin lesions and that there is a significant inflammatory response in mice with psoriasis and T2DM.

### 3.3 Evaluation of STING‐IRF3 pathway in psoriasis and T2DM animal model

**FIGURE 3:**
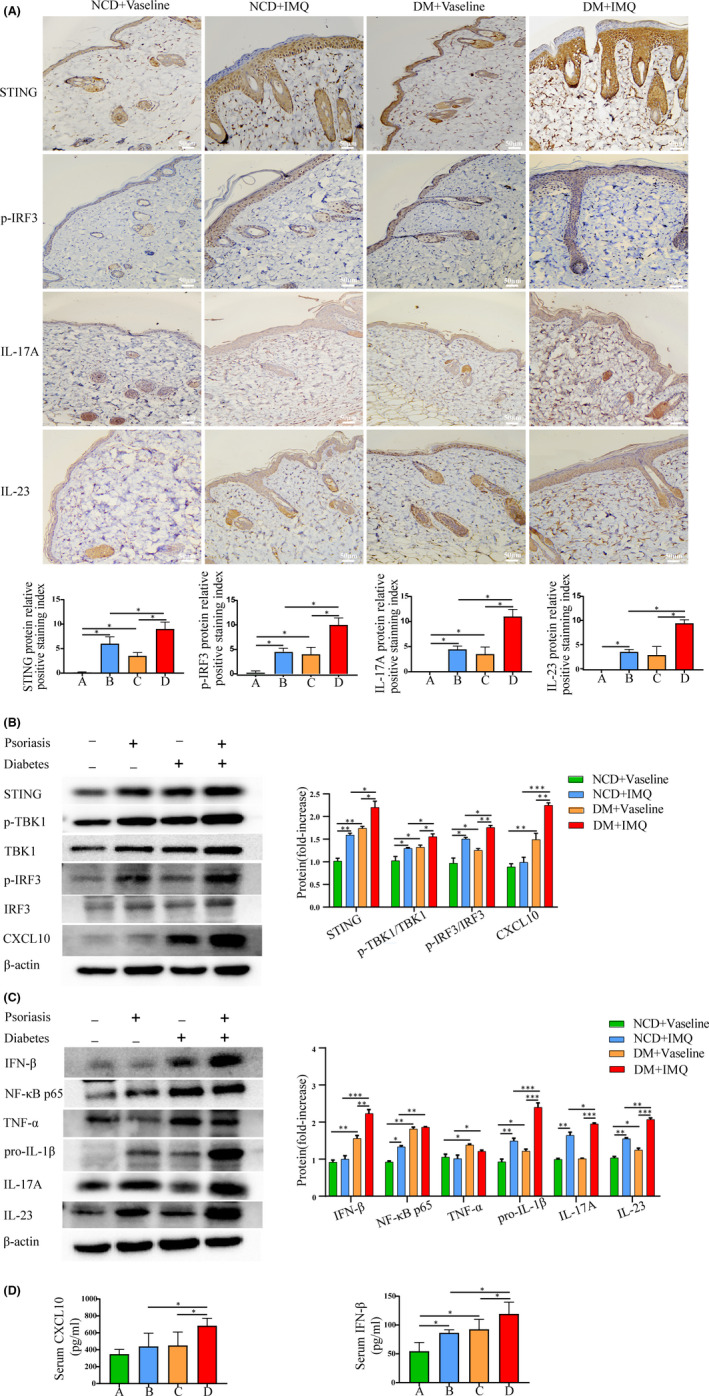
Evaluation of STING‐IRF3 pathway in psoriasis and T2DM animal model. Group A, NCD + Vaseline, Group B, NCD + IMQ, Group C, DM + Vaseline, Group D, DM + IMQ. (A) The protein levels of STING, p‐IRF3, IL‐17A and IL‐23 in the skin tissue were measured by immunohistochemistry (×200). (B, C) The protein levels of STING, p‐TBK1/TBK1, p‐IRF3/IRF3 and CXCL10, and inflammatory cytokines IFN‐β, NF‐κB p65, TNF‐α, pro‐IL‐1β, IL‐17A and IL‐23 in skin tissue were measured by Western blotting. (D) Serum CXCL10 and IFN‐β of mice were measured by ELISA. Data are represented as means ± SEM (*n* = 6). *p < 0.05; **p < 0.01; ***p < 0.001; ****p < 0.0001; Scale bars, 50 µm

To explore the role of the STING‐IRF3 pathway in psoriasis combined with T2DM, we examined the expression of the STING‐IRF3 pathway in a mouse model of psoriasis with T2DM. Immunohistochemistry showed that the dermal relative protein expression levels of STING, phospho‐IRF3 (p‐IRF3), interleukin‐17A (IL‐17A) and IL‐23 in the back skin tissue of the mice were significantly higher in the IMQ‐induction group. The increase in the DM + IMQ group was higher than that in the NCD + IMQ group (Figure 3A). The results of Western blotting showed that the levels of STING, phospho‐TBK1/TBK1 (p‐TBK1/TBK1), phospho‐IRF3/IRF3 (p‐IRF3/IRF3), CXCL10 and inflammatory cytokines (IFN‐β, NF‐κB p65, TNF‐α, pro‐inflammatory cytokine interleukin‐1 beta [pro‐IL‐1β], IL‐17A and IL‐23) in the skin tissue of the mice were higher in the DM + IMQ group (Figure 3B,C). In addition, the ELISA results showed that the expression of CXCL10 and IFN‐β in the serum of the DM + IMQ group was significantly upregulated (Figure 3D). Therefore, these data indicate that the STING‐IRF3 pathway is activated in the dorsal skin tissue of diabetic mice with psoriasis and is involved in the inflammatory response.

### 3.4 Correlation of STING‐IRF3 pathway with mitochondrial damage

**FIGURE 4:**
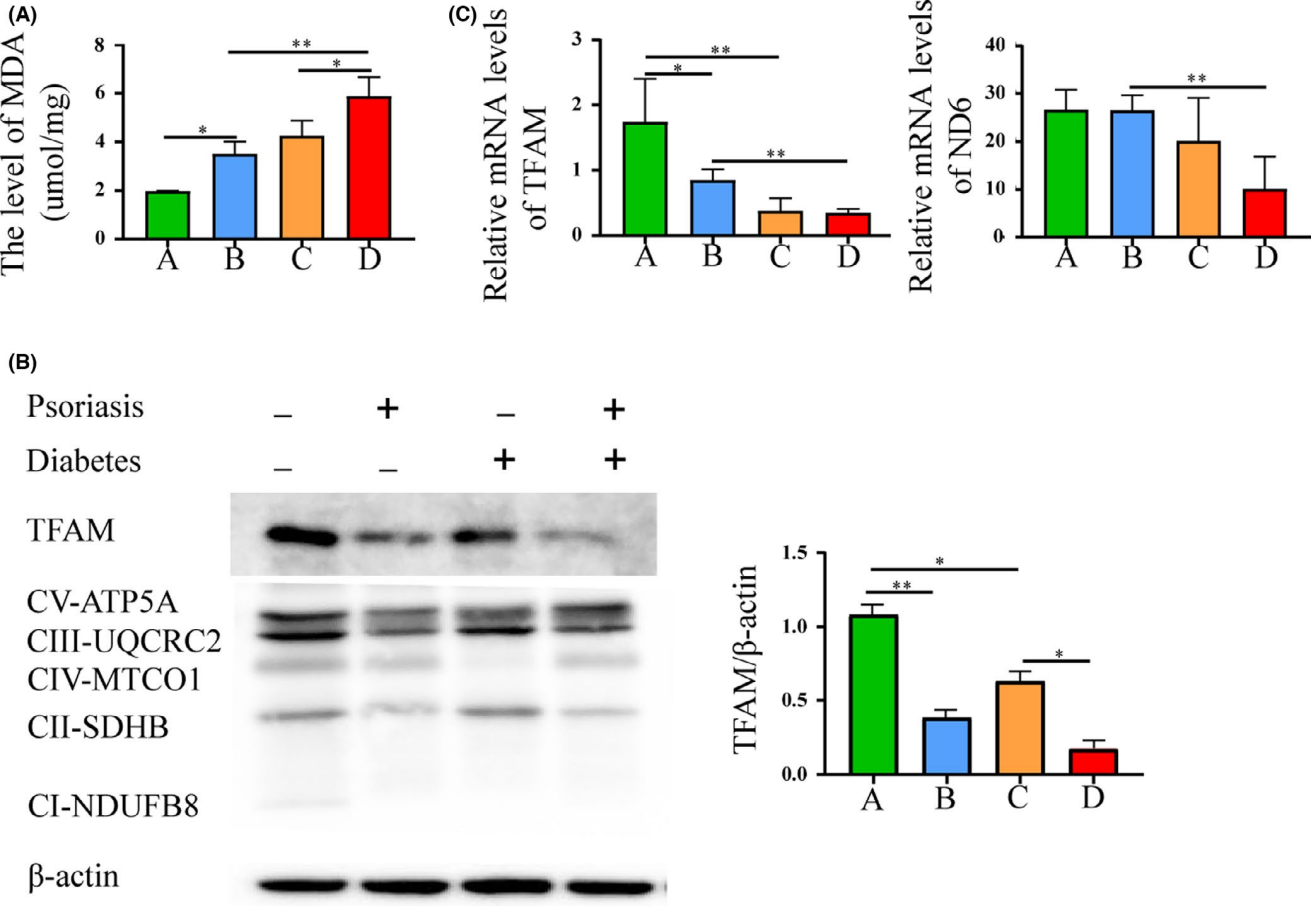
Correlation of STING‐IRF3 pathway with mitochondrial damage. Group A, NCD + Vaseline, Group B, NCD + IMQ, Group C, DM + Vaseline, Group D, DM + IMQ. (A) The level of oxidative stress was measured by lipid peroxidation (MDA) assay. (B) The level of TFAM and mitochondrial OXPHOS proteins (CV‐ATP5A, CIII‐UQCRC2, CIV‐MTCO1, CII‐SDHB and CI‐NDUFB8) in skin tissue were measured by Western blotting. (C) Total mRNA levels of mitochondrial‐related genes TFAM, ND6 in skin tissue were measured by RT‐qPCR. Data are represented as means ± SEM (*n* = 6). *p < 0.05; **p < 0.01; ***p < 0.001; ****p < 0.0001

Previous studies have suggested that mitochondrial damage activates STING.^20^, ^27^ To understand the possible role of the STING‐IRF3 pathway involved in the development of psoriasis with T2DM, we examined oxidative stress levels and expression levels of mitochondria‐related proteins and genes in the mouse model of psoriasis with diabetes. Malondialdehyde was used as a biomarker for oxidative stress levels. The lipid peroxidation assay showed that the level of MDA in the DM group was markedly higher than that in the NCD group, and that the DM + IMQ group was significantly elevated (Figure 4A). We further examined the mitochondrial function. Western blot analysis showed lower mitochondrial transcription factor A (TFAM) and oxidative phosphorylation of oxidative phosphorylation system (OXPHOS) proteins (CV‐ATP5A, CIII‐UQCRC2, CIV‐MTCO1, CII‐SDHB and CI‐NDUFB8) in the skin tissue of diabetic mice, especially in the DM + IMQ group (Figure 4B). The reverse transcription qPCR (RT‐qPCR) results showed that the relative mRNA levels of TFAM and nicotinamide adenine dinucleotide (NADH) dehydrogenase subunit 6 (ND6) in the skin tissue of diabetic mice were downregulated and the levels in the DM + IMQ group decreased significantly (Figure 4C), suggesting that diabetes combined with psoriasis may aggravate mitochondrial oxidative damage. Together, our data show that diabetes and psoriasis can induce mitochondrial damage, and it is speculated that mitochondrial damage is the cause of activation of the STING‐IRF3 pathway.

### 3.5 Inhibition of STING‐IRF3 pathway in diabetic mice with psoriasis

**FIGURE 5:**
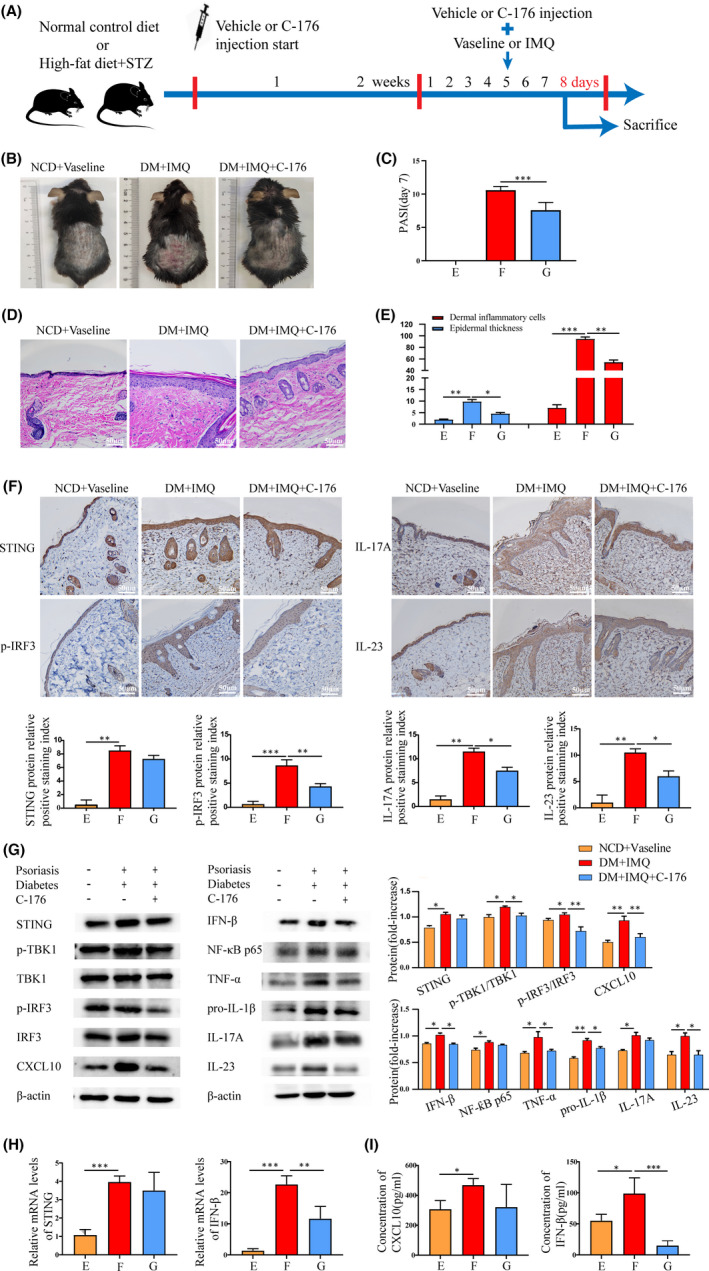
Inhibition of STING‐IRF3 pathway in diabetic mice with psoriasis. Group E, NCD + Vaseline, Group F, DM + IMQ, Group G, DM + IMQ + C‐176. (A) Experimental procedure for the STING inhibitor: C‐176. After 7 days of intervention with IMQ, (B) visual changes in the skin on the back of each group. (C) Comparison of PASI scores of back skin of mice in each group. (D) Histopathological changes were observed under light microscope after HE staining (×200). (E) The epidermal thickness and the number of inflammatory cells in the dermis were analysed by Image‐Pro Plus 6.0 software (×200). (F) Protein levels of STING, p‐IRF3, IL‐17A and IL‐23 in skin tissue were measured by immunohistochemistry (×200). (G) Protein levels of STING, p‐TBK1/TBK1, p‐IRF3/IRF3 and CXCL10, and inflammatory cytokines IFN‐β, NF‐κB p65, TNF‐α, pro‐IL‐1β, IL‐17A and IL‐23 in skin tissue were measured by Western blotting. (H) Total mRNA levels of STING and IFN‐β in skin tissue were measured by qRT‐PCR. (I) Serum CXCL10 and IFN‐β of mice were measured by ELISA. Data are represented as means ± SEM (*n* = 6 mice in each group). *p < 0.05; **p < 0.01; ***p < 0.001; ****p < 0.0001. Scale bars, 50 µm

To explore the therapeutic potential of inhibiting the STING pathway in mice with psoriasis and diabetes, we examined the effect of the newly reported STING‐specific inhibitor, C‐176. The experimental procedure for the STING inhibitor is shown in Figure 5A. After 2 weeks of advanced intervention with C‐176, the degree of erythema, scaliness and increased skin thickness in the DM + IMQ + C‐176 group was significantly improved. Additionally, the PASI in the DM + IMQ + C‐176 group was distinctly lower than that in the DM + IMQ group (Figure 5B,C). Haematoxylin and eosin staining then showed that the epidermal tissue thickness of mice reduced, and the number of inflammatory cells infiltrated in the dermis decreased in the DM + IMQ + C‐176 group (Figure 5D,E), suggesting that C‐176 can ameliorate inflammatory responses in diabetic mice with psoriasis. In addition, after C‐176 treatment, immunohistochemistry showed that the relative protein expression levels of p‐IRF3, IL‐17A and IL‐23 in the back skin tissue of mice in the DM + IMQ + C‐176 group were significantly lower than those in DM + IMQ group (Figure 5F). Western blotting results showed that the levels of STING‐IRF3 pathway downstream proteins (p‐TBK1/TBK1, p‐IRF3/IRF3 and CXCL10), and inflammatory cytokines (IFN‐β, NF‐κB p65, TNF‐α, pro‐IL‐1β, IL‐17A and IL‐23) in the skin tissue of mice were significantly downregulated in the DM + IMQ + C‐176 group when compared with the DM + IMQ group (Figure 5G). The RT‐qPCR results showed that the relative mRNA levels of IFN‐β in the skin tissue of the DM + IMQ + C‐176 group were also significantly reduced (Figure 5H). Additionally, the expression of CXCL10 and IFN‐β in the serum of the DM + IMQ + C‐176 group was markedly downregulated compared with the DM + IMQ group (Figure 5I), suggesting that C‐176 reduces the inflammatory response by inhibiting the activation of STING. Therefore, these data indicate that C‐176 inhibits the activation of the STING‐IRF3 pathway and ameliorates the inflammatory response in diabetic mice with psoriasis.

### 3.6 STING‐IRF3 pathway in skin samples from patients with psoriasis and T2DM

**FIGURE 6:**
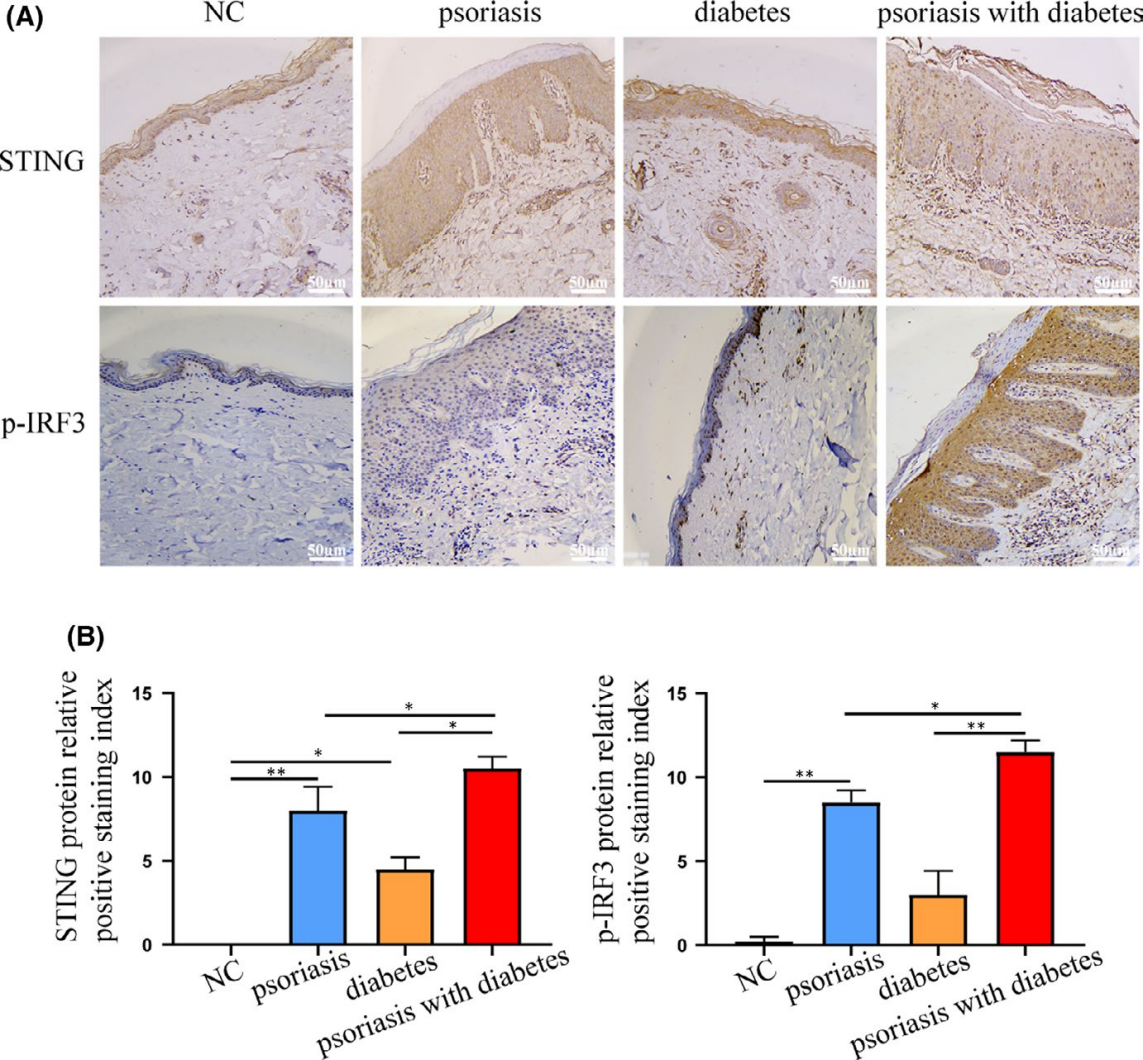
STING‐IRF3 pathway in skin samples from patients with psoriasis and T2DM. (A, B) Protein levels of STING, p‐IRF3 in the skin tissue of patients were measured by immunohistochemistry (×200). Data are represented as means ± SEM. *p < 0.05; **p < 0.01; ***p < 0.001; ****p < 0.0001. Scale bars, 50 µm

Figure 6A,B shows that the STING‐IRF3 pathway was expressed in the skin tissue of patients with psoriasis, T2DM or both. The protein expression levels of STING and p‐IRF3 were increased in the skin tissue of three kinds of patients, especially within the psoriasis with diabetes group when compared with healthy skin tissue samples.

## 4 DISCUSSION

Psoriasis and diabetes share the same pathophysiologies, in which genes, inflammation, environment and insulin resistance play crucial roles. People with psoriasis have an elevated risk of developing diabetes and vice versa.^28^ The concomitant presence of psoriasis and diabetes creates a complex pathological environment that poses great challenges for clinical treatment. At present, traditional systemic medications methotrexate, acitretin, cyclosporine and biologics anti‐TNFα, anti‐IL‐12/23, anti‐IL‐17 are the main treatment options for psoriasis. However, there is no consistent evidence to support the effectiveness of these drugs in the treatment of psoriasis with diabetes.^29^ In addition, researches on the clinical effects of metformin, GLP‐1 analogues and other antidiabetic medications on psoriasis have also yielded a diverse result, and some clinical studies have shown that these drugs cannot alleviate the severity of psoriasis.^30^ Therefore, it is urgent to explore common targets to find effective and alternative therapy to reduce health burden of psoriasis with diabetes. In this study, we provided both in vitro, in vivo and clinical evidence comprehensively confirming the involvement of STING‐IRF3 pathway in the pathological process of psoriasis and diabetes. The highlight of the study is that it is the first time to confirm the relationship between STING‐IRF3 pathway and psoriasis combined with diabetes, which broadens the understanding of the treatment of psoriasis with diabetes.

In the present study, we found that the STING‐IRF3 pathway was activated in HaCaT cells treated with PA and IMQ, as well as in the skin tissue of diabetic mice with psoriasis. The expression levels of STING and p‐IRF3 were also significantly upregulated in the skin tissue samples of patients with psoriasis and diabetes. We found that the level of MDA was increased in diabetic mice with psoriasis, whereas TFAM, OXPHOS‐related proteins and the expression of mitochondrial‐related genes were significantly decreased. After STING inhibitor C‐176 treatment, the infiltration rate of inflammatory cells in the back skin tissue of mice was reduced. The degree of skin tissue thickening and inflammatory cytokine levels were also reduced. Mechanistically, in the highly inflammatory environment of psoriasis combined with T2DM, oxidative stress damage causes mitochondrial dysfunction, activates the STING‐IRF3 pathway, and further aggravates the body's inflammation. Taken together, our data showed that the STING‐IRF3 pathway is involved in the inflammatory response of psoriasis combined with T2DM. Furthermore, mitochondrial damage might induce activation of the STING‐IRF3 pathway. Therefore, inhibition of this pathway is expected to be a therapeutic target for the treatment of diabetes and psoriasis.

Stimulator of interferon genes is an ER membrane protein that plays an important role in antiviral and innate immune responses. Stimulator of interferon genes connects upstream DNA sensors with downstream IRF‐3 and NF‐κB pathways and induces the expression of type I interferon and inflammatory molecules.^22^, ^31^, ^32^ Recently, it was reported that the STING‐IRF3 pathway can be activated by ER stress^19^ and mitochondrial damage.^20^, ^27^, ^33^ Additionally, IMQ has been reported to alter intracellular redox states. The inhibition of mitochondrial complex I by IMQ can induce the production of a large number of reactive oxygen species (ROS), damage mitochondria and disrupt mitochondrial balance.^34^ In our study, IMQ cream was used to induce skin damage in diabetic mice. In addition, there is growing evidence indicating that mtDNA escaping from stressed mitochondria can activate inflammasomes and participate in the cGAS‐STING pathway to induce interferon production.^27^, ^35^ Previous studies have shown that^36^ in keratinocytes, oxidative stress in a high‐glucose environment can cause ROS overload which triggers mitochondrial dysfunction, accelerate TFAM depletion, and mtDNA fragmentation.^20^ The abnormally packaged mtDNA escapes from the mitochondria to the cytoplasm and is recognized by the DNA sensor cGAS which then activates the STING‐IRF3 pathway, thus regulating immunity and metabolism.^36^ This is consistent with the results of our study showing that the STING‐IRF3 pathway was involved in the inflammatory response in mice with psoriasis and T2DM. Simultaneously, by using MDA as a biomarker for oxidative stress levels, we discovered that the level of MDA increased in the back skin tissue of diabetic mice with psoriatic lesions induced by IMQ. Using TFAM as a mtDNA biomarker, we also found that the expression of TFAM, mitochondrial OXPHOS proteins and ND6 was significantly reduced, suggesting that psoriasis with diabetes induces mitochondrial oxidative damage. Therefore, the STING‐IRF3 pathway may be involved in the oxidative stress response induced by psoriasis combined with diabetes through mitochondrial damage.

A growing number of studies have shown that the STING‐IRF3 pathway is involved in the regulation of immunity and metabolism. Hu et al.^37^ found that STING, p‐IRF3 and downstream protein IFN‐β were significantly upregulated in the PA‐induced insulinoma cell line (INS‐1) and pancreatic islets of db/db mice by Western blotting. STING or IRF3 knockouts can then improve inflammation and apoptosis induced by PA and reverse impaired insulin synthesis. The STING‐IRF3 pathway has also been shown to play an important role in pancreatic β‐cell lipotoxicity. However, the role of the STING‐IRF3 pathway in psoriasis with diabetes has not been reported. TANK‐binding kinase 1 is a direct downstream substrate that can be activated by STING.^38^ Interleukin‐17 and IL‐23 levels are elevated in the highly inflammatory environment of psoriasis complicated with diabetes. Studies have found that IL‐17 induces TBK1 activation with the assistance of tumour necrosis factor receptor‐associated factor 6 (TRAF6).^39^ In our study, we found that the expression levels of STING and p‐IRF3 were increased in the skin tissue of diabetic mice with IMQ‐induced psoriasis skin damage. The expression of the downstream proteins p‐TBK1/TBK1, p‐IRF3/IRF3 and CXCL10, and inflammatory cytokines IFNβ, NF‐κB P65, TNF‐α, IL‐1β, IL‐17A and IL‐23 were significantly upregulated. These data indicate that the STING‐IRF3 pathway is involved in the inflammatory response in psoriasis with T2DM. Furthermore, we studied the role of the STING‐specific inhibitor C‐176 in diabetic mice with psoriasis. C‐176 blocks palmitoylation induced by STING activation. Its assembly into polymer complexes in the Golgi apparatus is blocked, subsequently inhibiting downstream signal transduction. The pharmacological inhibition of STING significantly inhibited the activation of the STING downstream pathway, the infiltration of inflammatory cells in the dorsal skin tissue of mice was reduced, and the expression of downstream proteins and inflammatory factors were also significantly downregulated. C‐176 has a robust anti‐inflammatory effect.^40^ In accordance with this, it has been previously reported that STING inhibitor C‐176 could inhibit the activation of the STING pathway and ameliorate kidney fibrosis and inflammation in a model of kidney disease induced by folic acid (FA).^41^ Therefore, appropriate downregulation of the STING‐IRF3 pathway may be a new therapeutic target for individuals with psoriasis and diabetes.

Notably, although severe forms of psoriasis are more frequent in male versus female, there is no consensus on sex influence in the risk of diabetes in patients with psoriasis. A recent retrospective cohort study from Canada showed that both sexes have an increased risk of DM with psoriasis severity, whereas there were gender differences in prevalence across age groups.^42^ However, in our animal model studies, only male mice were used, and it is not very clear if the case was similar for the human samples. Therefore, further studies on the relationship between sex differences in psoriasis and diabetes and this pathway need to be further ruled out.

In summary, we have confirmed for the first time that the STING‐IRF3 pathway is involved in the inflammatory response in the manifestation of psoriasis with T2DM. In this study, we concluded that STING‐IRF3 pathway activation plays a key role in mediating the development of psoriasis with diabetes. Limiting STING activity might have an ameliorating effect on the development of psoriasis with diabetes. However, there are some limitations in our study, such as a low number of animals, a low number of patients with double pathology, a lack of detection of cytokines or chemokines in the serum of patients. Nonetheless, our preliminary studies enrich the understanding of the treatment of psoriasis with diabetes and provide direction for the development of therapeutic agents for these diseases.

## CONFLICT OF INTEREST

The authors confirm that there are no conflict of interest.

## AUTHOR CONTRIBUTIONS

**Chen Pin:** Conceptualization (lead); Supervision (lead). **Li Xiaohong:** Methodology (supporting); Writing – original draft (lead). **Zhang Zhenting:** Methodology (supporting); Writing – original draft (supporting). **Yu Yunjie:** Visualization (lead); Writing – original draft (supporting). **Cai Wei:** Formal analysis (supporting); Methodology (supporting). **Xu Xiangjin:** Conceptualization (supporting); Writing – review & editing (supporting). **Xie Kun:** Methodology (supporting); Supervision (supporting). **Lin Xin:** Data curation (supporting); Methodology (supporting). **Lin Lu:** Data curation (supporting); Methodology (supporting). **Lu Jun:** Conceptualization (lead); Writing – review & editing (supporting).

## Supporting information

Table S1

Table S2

Table S3

## Data Availability

The original contributions presented in the study are included in the article/supplementary material, further inquiries can be directed to the corresponding author.
