# Supplementary material for: Activation of the STING‐IRF3 pathway involved in psoriasis with diabetes mellitus
Source: J Cell Mol Med. 2022 Feb 17;26(8):2139–51. doi: 10.1111/jcmm.17236 (PMC8995451; doi:10.1111/jcmm.17236)
Supplement: Supplementary file 1 — Table S1 [file JCMM-26-2139-s002.docx]

**Supplementary Table S1**

**S1**-**1:** **The primary antibodies used for immunohistochemistry^[[1]](#footnote-1)^.**

| Antibody | Company | Catalog# | Source | MW (KDa) |
| --- | --- | --- | --- | --- |
| STING  p-IRF3  IL-17A  IL-23 | Proteintech, USA  CST, USA  abcam, USA  abcam, USA | 19851-1-AP  29047S  ab79056  ab45420 | Rabbit  Rabbit  Rabbit  Rabbit | 35-40  45-55  18  24 |

**S1**-**2: The primary antibodies used for** **Western blot^[[2]](#footnote-2)^.**

| Antibody | Company | Catalog# | Source | MW (KDa) |
| --- | --- | --- | --- | --- |
| STING  p-TBK1  TBK1  p-IRF3  IRF3  CXCL10  IFN-β  NF-κB p65  TNF-α  pro-IL-1β  IL-17A  IL-23  TFAM  OXPHOS  β-actin | Proteintech, USA  CST, USA  CST, USA  CST, USA  CST, USA  ABclonal, China  ABclonal, China  CST, USA  ABclonal, China  ABclonal, China  abcam, USA  abcam, USA  ABclonal, China  abcam, USA  CST, USA | 19851-1-AP  5483S  3504S  29047S  11904S  A19138  A1575  8242S  A11534  A16288  ab79056  ab45420  A13552  ab110413  3700S | Rabbit  Rabbit  Rabbit  Rabbit  Rabbit  Rabbit  Rabbit  Rabbit  Rabbit  Rabbit  Rabbit  Rabbit  Rabbit  Mouse  Mouse | 35-40  84  84  45-55  50-55  10  22  65  28  31  18  24  25  /  45 |

1. STING: stimulator of interferon genes; p-IRF3: phosphorylated interferon regulatory factor 3; IL-17A: interleukin 17A; IL-23: interleukin 23; CST: Cell Signaling Technology. [↑](#footnote-ref-1)
2. STING: stimulator of interferon genes; p-TBK1: phosphorylated TANK-binding kinase 1; p-IRF3: phosphorylated interferon regulatory factor 3; CXCL10: interferon gamma-induced protein 10; IFN-β: interferon beta; NF-κB: nuclear factor kappa B; IL-1β: interleukin-1 beta; IL-17A: interleukin 17A; IL-23: interleukin 23; TFAM: mitochondrial transcription factor A; OXPHOS: oxidative phosphorylation system proteins (CV-ATP5A 55KDa, CIII-UQCRC2 48KDa, CIV-MTCO1 40KDa, CII-SDHB 30KDa, and CI-NDUFB8 20KDa); CST: Cell Signaling Technology. [↑](#footnote-ref-2)
