# Supplementary material for: Activation of the STING‐IRF3 pathway involved in psoriasis with diabetes mellitus
Source: J Cell Mol Med. 2022 Feb 17;26(8):2139–51. doi: 10.1111/jcmm.17236 (PMC8995451; doi:10.1111/jcmm.17236)
Supplement: Supplementary file 2 — Table S2 [file JCMM-26-2139-s003.docx]

**Supplementary Table S2: Primers used for Real-time PCR.**

| Gene | primer |
| --- | --- |
| Mouse-STING forward  Mouse-STING reverse  Mouse-IFN-β forward  Mouse-IFN-β reverse  Mouse-TFAM forward  Mouse-TFAM reverse  Mouse-ND6 forward  Mouse-ND6 reverse  Mouse-β-actin forward  Mouse-β-actin reverse | AGTCCCTAAGCATGCTCCTG  TGGGGTCAACTACACTCAGG  CTACAGGGCGGACTTCAAGA  AGTGGAGAGCAGTTGAGGAC  GAGCAGCTAACTCCAAGTCAG  GAGCCGAATCATCCTTTGCCT  TTAGCATTAAAGCCTTCACC  CCAACAAACCCACTAACAAT  TCCAGCCCTCTTTCATTGGT  TGATCTTCATGGTGCTGGGA |
