# Supplementary material for: Activation of the STING‐IRF3 pathway involved in psoriasis with diabetes mellitus
Source: J Cell Mol Med. 2022 Feb 17;26(8):2139–51. doi: 10.1111/jcmm.17236 (PMC8995451; doi:10.1111/jcmm.17236)
Supplement: Supplementary file 3 — Table S3 [file JCMM-26-2139-s001.docx]

**Supplementary Table S3: Demographic and Baseline Clinical Characteristics^[[1]](#footnote-1)^.**

| Characteristics | NC  (n=6) | Psoriasis  (n=5) | Diabetes  (n=5) | Psoriasis with  diabetes(n=6) |
| --- | --- | --- | --- | --- |
| Age, years  Men, n (%)  Women, n (%)  PASI  DLQI  Weight, kg  BMI, kg/m^2^  FBG, mmol/L  HbA1c, %  FCP, ng/ml  FINS, µU/mL  HOMA-IR | 46.92±4.21  4 (66.7)  2 (33.3)  /  /  61.14±4.72  22.84±2.16  5.17±0.56  5.40±0.44  0.83±0.13  8.17±2.09  1.73±0.32 | 25.80±5.27  5 (100)  0 (0)  8.16±2.12  12.80±4.49  68.40±5.08  23.85±0.92  4.78±0.53  5.28±0.26  1.22±0.58  10.14±4.06  2.15±0.86 | 59.00±8.25  3 (60)  2 (40)  /  /  69.4±6.77  24.9±0.81  9.40±1.89  8.26±1.93  0.94±0.25  11.61±3.81  5.04±2.16 | 55.83±2.61  4 (66.7)  2 (33.3)  14.30±6.80  19.17±6.67  72.83±10.43  27.45±3.98  9.76±2.26  8.45±1.21  2.93±1.61  16.86±6.25  6.97±2.13 |

1. PASI: psoriasis area severity index; DLQI: dermatology life quality index; BMI: body mass index; FBG: Fasting blood glucose; HbA1c: glycosylated hemoglobin A1c; FCP: Fasting C-peptide; FINS: Fasting insulin; HOMA-IR: homeostasis model assessment of insulin resistance.

   The date is presented by the means ± SD. [↑](#footnote-ref-1)
